# Productivity, carbon sequestration and species diversity in virgin and secondary meadow steppes of the Bashkir Cis-Urals

**DOI:** 10.1038/s41598-025-02493-y

**Published:** 2025-05-19

**Authors:** Nikolay Fedorov, Pavel Shirokikh, Svetlana Zhigunova, Elvira Baisheva, Mikhail Komissarov, Albert Muldashev, Dilara Gabbasova, Milyausha Akhmetova, Ilshat Tuktamyshev, Ilnur Bikbaev, Galina Shendel, Davut Gulov, Mikhail Aivazyan, Vazir Gimazetdinov, Vasiliy Martynenko

**Affiliations:** 1 Ufa Institute of Biology, Ufa Federal Research Centre of the Russian Academy of Sciences, Ufa, 450054 Russia; 2 Ufa State Petroleum Technological University, Ufa, 450062 Russia

**Keywords:** Soil carbon, Secondary steppes, Agricultural use, Carbon content, Agroecology, Climate-change ecology, Ecosystem ecology, Environmental impact, Agroecology, Community ecology, Ecosystem ecology, Grassland ecology

## Abstract

Steppes are of great importance for the global biogeochemical cycle and are characterized by high economic value. Carbon stocks in the soil of flat steppe landscapes are about one-fourth of the total carbon deposited in global soils. However, improper methods of pasture management, especially overgrazing, have a serious negative impact on the structure and functioning of steppes. The aim of this study is to analyze carbon accumulation in virgin and secondary meadow steppes in the Bashkir Cis-Urals (Russia) depending on various methods of agricultural use. The data were collected on 10 sample plots laid on cropland, as well as in secondary and virgin meadow steppes. It was found that secondary meadow steppes on fallow lands abandoned for about 20–45 years are close to virgin steppes in terms of the dominant species composition but differ by low floristic diversity, a different proportion of steppe specialist species and lower root phytomass (60–100% lower than in the virgin steppe). The phytomass of all fractions of plant matter was the highest in virgin steppe. Under moderate agricultural use (occasional and moderate haymaking or grazing), the succession goes towards the restoration of steppe community structure and soil organic carbon content. Intensive grazing slows down the restorative succession and reduces the organic carbon content in the soil. Compared with the meadow steppes located at the foot and the lower part of the hill, the steppes of upper and middle parts of the same slope have a high stock of above-ground phytomass but contain less carbon in the soil due to water erosion.

## Introduction

Steppes are a zonal vegetation type of Eurasia located in Central and Eastern Europe, in the south of Western Siberia, in Central Asia and mountainous regions. The steppe biome is of great importance for the global biogeochemical cycle and hosting biodiversity, and it is characterized by high economic value and anthropogenic development^1,2^. Steppe vegetation is formed mainly by perennial grasses with a strong root system. A share of roots can reach 87% of the total biomass and 60–80% of the carbon contained in the phytomass of steppe communities^3,4^ and the share of roots in the total biomass largely determines the soil-forming effect of steppe communities^5^. Steppes account for about 27% of the total land area, and carbon sequestration in these ecosystems play a significant role in the global carbon cycle^6,7^.

The most fertile soil types of Eurasia – chernozems and kastanozems – were formed in steppe ecosystems. However, improper methods of pasture management, especially overgrazing, have had a serious negative impact on the structure and functioning of these ecosystems^8–10^. Currently, about 50% of the world’s natural pasture land has been degraded^10,11^.

Pasture degradation and changes in land use due to overgrazing alter the composition and structure of steppe plant communities^12,13^ reduce species diversity and primary productivity^14^, as well as soil organic carbon and nutrient availability^8^. Thus, grazing intensity has the potential to alter soil structure, function and organic carbon storage capacity and can significantly alter pasture carbon stocks^15,16^. In this article, secondary (restored) steppes are understood as a post-agrogenic successional steppe ecosystems that are restored on abandoned arable lands (usually, over 20 years or more) and in which grass species typical of regional virgin steppes dominate^17^.

As soil organic carbon (SOC; or soil C_org_) has a major influence on soil physical structure and a range of ecosystem services (e.g., nutrient retention, water storage, pollutant attenuation), its reduction could lead to reduced soil fertility and consequently, land degradation^15,18^. An analysis of pools of organic carbon in different types of soils in Russia showed, that the steppe soils have the maximum carbon stocks (150–180, sometimes up to 210 t/ha) accumulated in the mineral strata of the most humus-rich varieties of chernozems^19^. In the Southern Area of the East European Plain, the steppe Chernozems even 145 years ago had a SOC content of up to 4%, then the Chernozems in the forest-steppe zone, which used to have habitats with a SOC content of 4–7%, occupied the largest areas, and have now lost 30–40% of the original values in the 0–50 cm layer due to the human impact^20^. These effects may also be amplified if the rate of SOC loss increases due to climate change^16,21^, and thereby reducing the function of steppes as greenhouse gas (GHG) sinks^22^. As a consequence, grassland soils could become a source rather than a sink for GHG emissions^16,23–25^. In general, these effects are common to all climatic and geo-graphical zones, although the extent to which grazing affects SOC depends on climate^16^. Negative management effects decrease with increasing mean annual temperature and mean annual precipitation in the temperate zone^26^.

A considerable part of Russian steppes is occupied by agricultural lands, while natural virgin steppes have been preserved in relatively small areas, mainly with rugged terrain, poor soils and low aridity index values^2^. The total area of natural steppe ecosystems in Russia is estimated at 200,000 km^2^, comprising less than 12% of the total steppe-covered areas of country. In the last decade of the 20 th century, significant areas of Russian arable land were withdrawn from agricultural activities. Under favorable conditions, it took 15–30 years to restore steppe ecosystems close to virgin steppes that existed before plowing. When restoring steppe vegetation, the amount of organic matter supplied into the soil increased, and the natural physicochemical properties and microbiological characteristics of soils were gradually restored^27^. In particular, soil horizons of fallow lands demonstrate the changes in their structure, density, air-and-water and hydrothermal regimes, and the increase in contents of carbon and mineral nutrients for plants^10,27,28^.

In the Republic of Bashkortostan, virgin and secondary meadow steppes are widespread in the forest-steppe and steppe zones, however, the carbon storage in these ecosystems has not been sufficiently studied. In the study area, vegetation productivity and carbon stock were studied only in one meadow steppe area, located on fallow land abandoned about 20 years ago^29^ The aim of this study is to analyze carbon accumulation in virgin and secondary meadow steppes in the Bashkir Cis-Urals depending on various methods of agricultural use and the position in the relief.

## Materials and methods

### Site description

The study area is located in the forest-steppe and steppe zones of the southern (Bashkir) Cis-Urals and partially includes two landscape regions: the Bugulma-Belebeevskaya Upland and the Pribelskaya Plain. The relief is a gently hilly plain. The region is densely populated, and the vegetation cover is human-modified. Zonal forest vegetation is represented by small forest areas dominated by oak (*Quercus robur* L.), linden (*Tilia cordata* Mill.) and birch (*Betula pendula* Roth), which are found mainly on hillsides. The native steppe vegetation is represented mainly by meadow steppes, most of which were plowed and used for agriculture in the past. Small fragments of undisturbed virgin steppe have survived only in areas inconvenient for plowing, mainly on steep hillsides. At the end of the 20 th century, as a result of economic reforms in Russia, the intensity of livestock grazing and the use of arable land decreased, and many croplands in the study area were abandoned and over time turned into secondary (restored) steppe.

Based on morphological properties, the soil cover of the study area is presented by chernozems^30^, with a “medium” thickness of humus-accumulative horizons (40–60 cm) and a sandy loam texture of the topsoil.

**Fig. 1:**
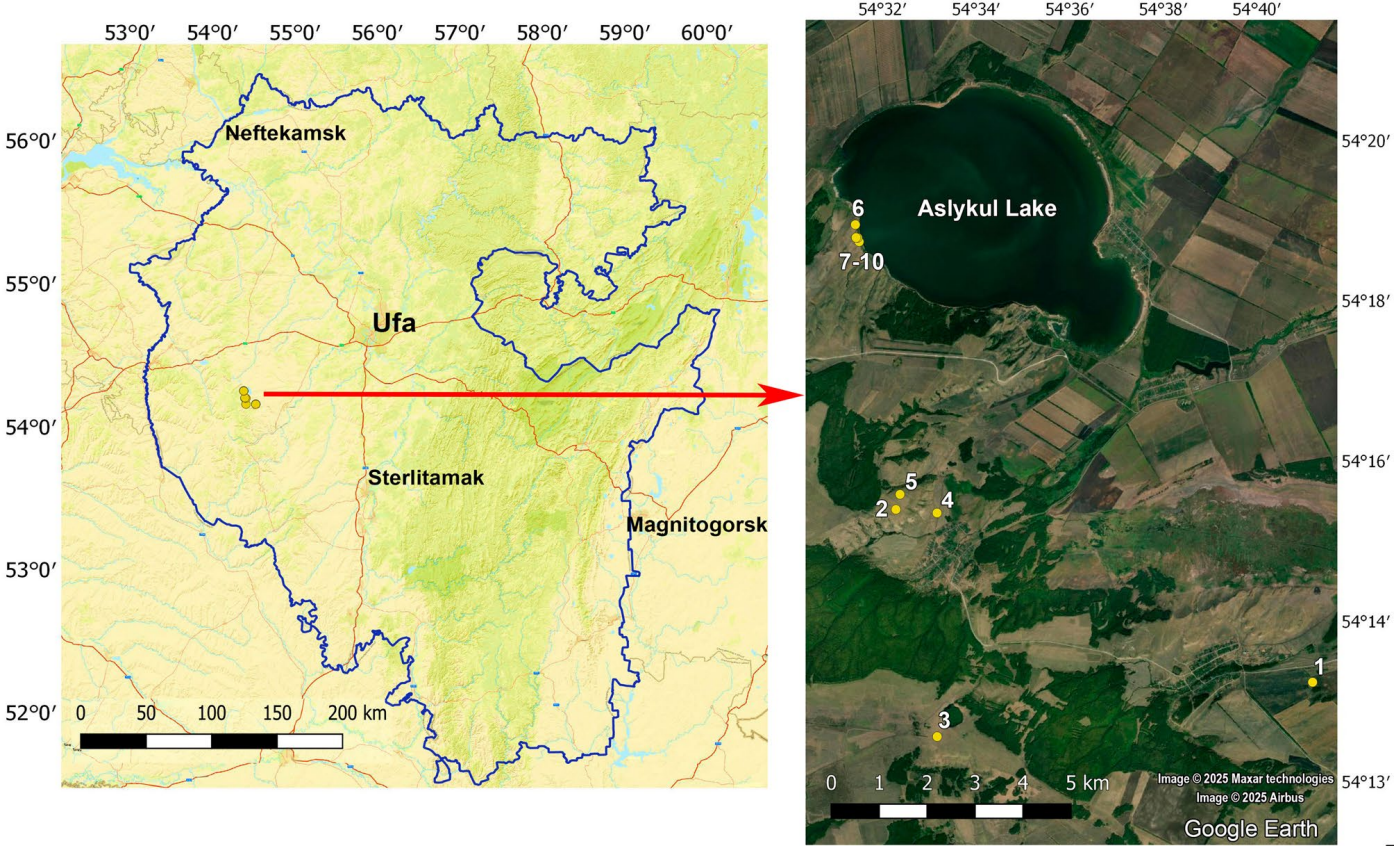
Location of the study area in the Republic of Bashkortostan. The sample plots are marked in yellow color.

The sample plots are located in the “Asly-Kul” Nature Park with an area of 47,500 ha, which was created in 1993 to protect the landscape complex of Aslykul Lake (23.5 km²), the largest in the Republic of Bashkortostan (Fig. 1).

The climate of the study area is continental, with insufficient moisture. The average annual temperature is + 3.3 °C; the average January temperature is − 14.2 °C; July is + 20 °C; the average depth of soil freezing by the end of winter is up to 90–130 cm. The average annual precipitation is 400 mm, the frost-free period lasts 125 days, and the vegetation period is 170 days. The melting of snow cover on leveled areas is observed in late March – early April.

In 2023–2024, weather conditions during the growing season varied significantly. Compared with the average long-term weather conditions for the Republic of Bashkortostan, 2023 was slightly dry, and 2024 was abnormally wet (Table 1). The air temperature in June, during the period of intensive plant growth, was slightly higher in 2024 than long-term observations.

**Table 1:** Monthly average precipitation and temperature in the periods preceding the start of the research.

	Precipitation, mm			Temperature, ºC		
Months	2023	2024	Average for 2019–2024	2023	2024	Average for 2019–2024
March	17.4	22.9	26.7	0.6	–4.6	–3.9
April	6.7	33.4	21.8	9.3	11.9	8.9
May	29.1	6.5	21.0	16.5	11.0	14.3
June	28.7	100.7	54.2	17.5	20.9	18.9
July	22.4	85.4	41.2	22.4	20.5	21.7

## Description of sample plots

**Fig. 2:**
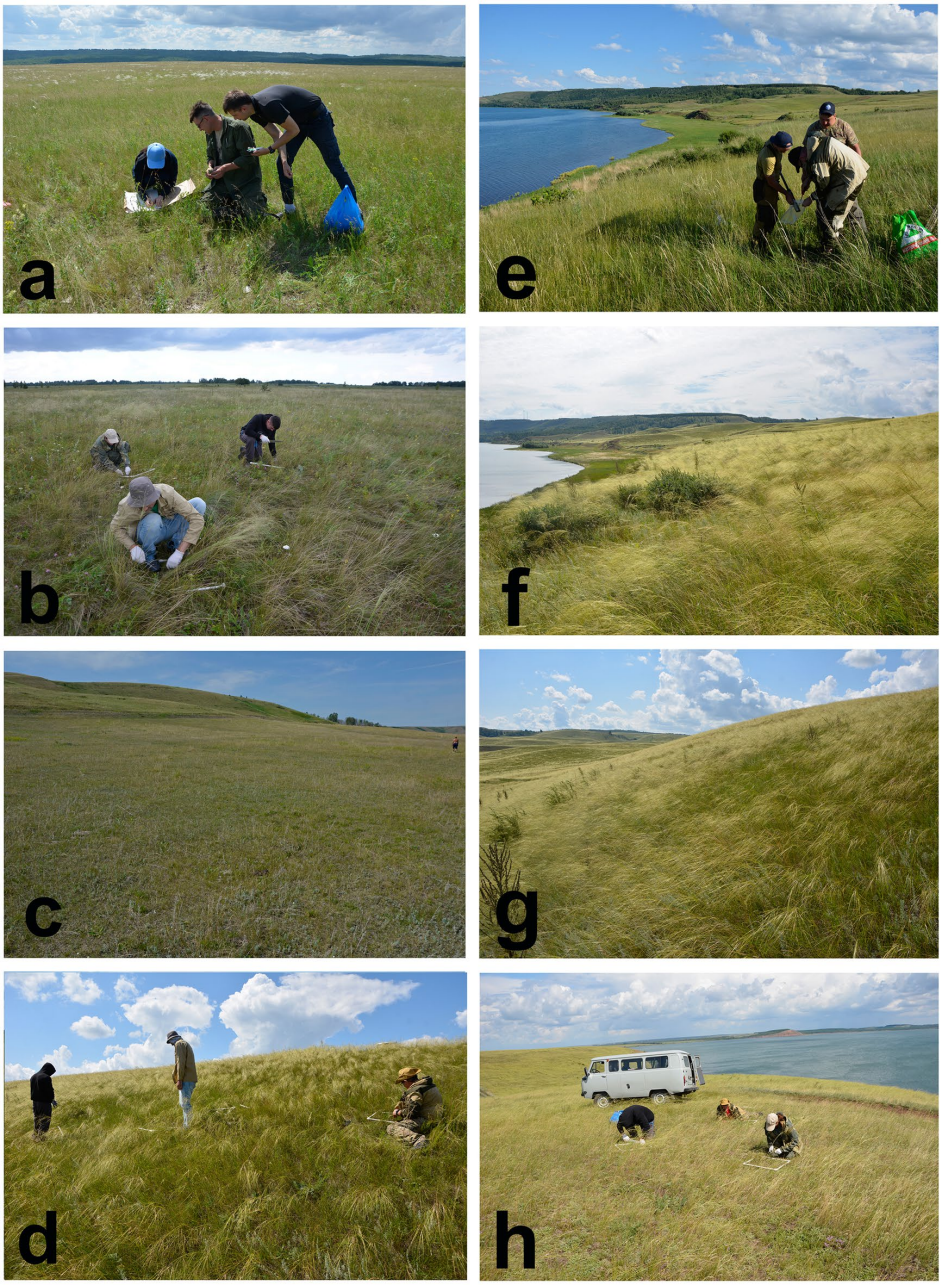
Investigated steppe communities: a – the secondary steppe with occasional haymaking (SP 2); b – the secondary steppe with moderate grazing (SP 3); c – the secondary steppe with heavy grazing (SP 4). d – virgin steppe with occasional grazing (SP 6). Position of the sample plots on the slope: e – foot (SP 7); f – lower part (SP 8); g – middle part (SP 9); h – upper part (SP 10).

In 2023–2024, 10 sample plots (SP) were laid: one on cropland (SP 1), four (SPs 2–5) in restored steppe, and five (SPs 6–10) in virgin steppe (Fig. 2). The characteristics of the sites where the sample plots were established are given in Table 2.

**Table 2:** The characteristics of the areas where the sample plots were established in the “Asly-Kul” nature park.

No. of sample plot	Current use	Exposure and slope steepness,º	Position on the slope	Year of observations
1	C	–	–	2023
2	OH	S, 2	–	2023
3	MG	–	–	2023–2024
4	HG	NE, 2	–	2023
5	OG	–	–	2023
6	OG	NE, 10	MP	2023–2024
7	OG	E, 12	F	2024
8	OG	E, 12	LP	2024
9	OG	E, 12	MP	2024
10	OG	E, 12	UP	2024

Current agricultural use: С – cropland, OH – occasional haymaking, OG – occasional grazing, MG – moderate grazing, HG – heavy grazing. Plot characteristics: R – flat plot (without inclination), S – southern exposition/slope, NE – north-eastern slope, E – eastern slope. Position on the slope: F – foot, LP – lower part, MP – middle part, UP – upper part. The length of the slopes with sample plots 7–10 is 150 m.

The sites with SPs 2–5 were previously plowed, and secondary steppe communities began to restore on them due to the process of natural regeneration of vegetation after the withdrawal of agricultural lands from the crop rotation. Within SPs 2–6, the square site sized 100 m^2^ were selected to study in detail composition and structure of the plant community. SPs 6–10 are located on steep slopes. SPs 7–10 were laid out in the foot, lower, middle and upper parts of the slope with a length of 150 m, to analyze the relationship between the stock of plant matter in steppe communities and the relief, which affects the moisture supply of the soil.

In the sample plots, agricultural use methods have differed during recent decades. SP 2 was influenced by episodic haymaking: the hay is mown on it in years with wet summers and not mown in years with dry summers. The sites with SPs 3–10 were subject to grazing pressure of varying intensity (Table 2).

## Estimation of the biomass of herbaceous plants and Mortmass

To analyze the productivity of above-ground phytomass of herbaceous plants during the period of their maximum development (at the end of June), we randomly set 20–25 square plots sized 50 × 50 cm on each of the sample plots. Within each square sample plot, the above-ground parts of herbaceous plants were cut, and the dead biomass was sampled. The dead biomass included both plant litter (dead decomposing plants) and dead parts of herbs that have not yet lost their connection with living plants. All samples were dried to an air-dry state and weighed with an accuracy of 0.01 g.

## Estimation of root phytomass

The root phytomass was estimated using the soil core method^31^. For this purpose, one core with a diameter of 5 cm was taken at each site in the 0–30 cm soil layer. Before analysis, soil and non-organic material were carefully washed away from roots by running tap water and were then manually separated from organic debris. All root samples were oven-dried at 60 **º**C to a constant mass, and their weights were measured using analytical scales (VLTE-150, Gosmeter, Russia) with an accuracy of 0.001 g.

## Analysis of the carbon content of the samples

Samples of live and dead biomass were ground with Vilitek cutting mills (VLM series) to a particle size of less than 0.5 mm. The finest parts of the roots were ground to a powder in porcelain mortars with liquid nitrogen. The carbon content in the samples was determined using a CHNS EA-3100 elemental analyzer (Eurovector, Pavia, Italy) in the Laboratory of Physical and Chemical Methods of Analysis (PCMA) at the Ufa Institute of Chemistry of UFRC RAS. The calculations of the quantitative content were provided by a special software package Weaver.

### Soil characteristic, sampling, and laboratory analysis

The soil profile pits were excavated in each plot with the following sizes: 3 m in length, 1 m in width, and to a depth of 0.5–0.7 m, where in most cases the illuvial horizon (B) is formed. The digging of soil profiles was caused by the need for soil type determination (using morphological description of each genetic horizon and further obtained data of agrichemical results) and sampling. Soil samples were taken from the topsoil (0–30 cm) layer using a small shovel. In order to increase the statistical reliability, the extra soil core samples (3 pcs within plot) were taken from the same depth (of 0–30 cm) using a hand sampler (JMC, Newton, MA, USA; inner diameter: 4.5 cm) via a stratified random scheme. The depth of soil sampling chosen corresponded to the studied underground phytomass layer^32^. Moreover, it was noted that SOC mostly presented in topsoil (0–30 cm), and in this layer, the largest changes in SOC content and other physicochemical properties are observed^33,34^.

The soil samples (~ 300 g) were collected in a plastic bag and then delivered to a laboratory. The stones and tree/plant roots were removed from the samples, then samples were air-dried to constant weight, ground in a mortar, and sieved through a 2 mm sieve for further analysis. The agrochemical properties were determined by standard methods^35^. In particular, the SOC in the soil samples was determined by the wet-combustion method according to Tyurin^36^ (direct analog of Walkley–Black method^37^) using a Specord M40 spectrophotometer (VEB Carl Zeiss, Jena, Germany). The available phosphorus (Pav) and exchangeable potassium (Kex) were extracted in 0.5 mol L^−1^ CH_3_COOH at a 1:2.5 soil/solution ratio by Chirikov method (topsoil is non-carbonate, with neutral pH).

The water-physical properties measurements were performed according to Vadyunina and Korchagina^38^ methodology. In particular, the structural-aggregate composition (dry sieving) was determined by using meshes with sizes of 10, 7, 5, 3, 1, 0.5 and 0.25 mm; the structural coefficient (Ks) as the main indicators in assessment/quality of soil aggregate composition was estimated according to the Eq. (1):

Ks = Σ (0.25–10 mm)/Σ (> 10, < 0.25 mm) (1).

Soil aggregate stability (wet sieving) was measured with using a Baksheev device (Vibrotehnic, Saint Petersburg, Russia); the SAS coefficient was calculated from the Eq. (2):

Ksas = Σw/Σd, (2)

where Σw – sum of aggregates > 0.25 mm under wet sieving (water-stable aggregates), Σd – sum of aggregates > 0.25 mm under dry sieving.

The particle size distribution was determined by “Laska-TD” laser diffraction analyzer (BioMedSystem, Saint Petersburg, Russia).

The SOC stocks were calculated based on SOC content and soil bulk density, particularly using the following formula (3):

SOC stocks (t/ha) = SOC content (%) × L (soil layer, cm) × bulk density (g/cm^3^). (3)

The determination/calculation of bulk density (mass of oven-dried soil ÷ total soil volume) was made in an established manner^38,39^. For this, the undisturbed soil samples were taken from cross-sectional profiles using metal cylinders (10 cm height and diameter), which were hammered in every 10 cm (until 30 cm).

## Results

### Floristic composition

In the communities described on SPs 2, 3, 5–10, the species typical for steppes and meadows (*Poa angustifolia* L., *Festuca pseudovina* Hack. ex Wiesb., *Stipa capillata* L., *Stipa pennata* L., *Fragaria viridis* Weston, *Agrimonia asiatica* Juz., *Euphorbia virgata* Waldst. & Kit.) have high projective cover. Comparative syntaxonomic analysis showed that these communities belong to the association *Poo angustifoliae-Stipetum pennatae* Yamalov et al. 2013, the order *Brachypodietalia pinnati* Korneck 1974 and the alliance *Cirsio-Brachypodion pinnati* Hadač & Klika in Klika & Hadač 1944, which unites the mesoxerophytic basiphilous grasslands of the subcontinental regions of Europe^40,41^. The communities of this association are widespread in various habitats of the forest-steppe zone of the South Ural region: northern slopes and tops of small mountains and hills, as well as on flat interfluve. These communities have high economic value and are often used as hayfields and pastures^42^.

As a result of intensive grazing, the plant community dominated by *Artemisia austriaca* Jacq., *Festuca pseudovina* Hack. ex Wiesb. and *Poa angustifolia* L. was formed on SP 4. These communities can be attributed to the class *Polygono-Artemisietea austriacae* Mirkin et al. in Ishbirdin et al. 1988, which unites communities of low-growing xerophytic plants of the steppe zone that are resistant to trampling and grazing^43^.

SPs 2–5 are located on relatively flat relief elements (Table 2), so it was assumed that the differences in the floristic structure of communities in these plots are associated with the peculiarities of their economical use. Table 3 shows that the projective cover and height of the herb layer of communities in SPs 2 and 5 are close to SP 6. Compared with SP 6 (the permanent steppe), the floristic composition of the communities from restored steppes of SPs 2–5 is characterized by lower indices of species diversity and species saturation per unit area, the absence or a smaller proportion of species typical for regional virgin meadow steppes (for instance, *Adonis vernalis* L., *Anemone sylvestris* L., *Dianthus versicolor* Fisch. ex Link, *Plantago urvillei* Opiz and some other species were not found in SPs 2–5). These differences increase with increasing grazing pressure. Thus, in comparison with SPs 5 and 3, the community in SP 4 has lower rates of the number of species, the projective cover and the height of the herb layer.

Weeds (*Artemisia austriaca* Jacq., *Carduus acanthoides* L., *Convolvulus arvensis* L., etc.) and weed-meadow species (*Verbascum lychnitis* L., *Salvia verticillata* L., *Taraxacum officinale* H. Wigg., etc.) make a significant contribution to the floristic composition of restored steppes. In the sample plots of the gentle agricultural use regime (SP 2 and SP 5), the proportion of these species in the total species richness is 24.2% and 23.9%, respectively. In areas with higher grazing intensity, the share of weeds and weed-meadow species increases (SP 3–31.5%, SP 4–43.6%). Thus, an increase in grazing intensity slows down the restoration succession and prevents the replacement of weeds by species typical of steppe vegetation. The time after the cessation of plowing (for SP 3–26 years, and for SP 4–45 years) does not play a significant role if the recovering steppe is under intensive grazing.

**Table 3:** Some characteristics of meadow steppe communities in the sample plots studied in 2023.

No. of sample plot	2	3	4	5	6
**Method of agricultural use**	occasional haymaking	moderate grazing	heavy grazing	occasional grazing	occasional grazing
**The projective cover of the herb layer**,** %**	60	70	40	65	60
**Mean height of the herb layer**,** cm**	25	25	15	25	30
**Number of species in a sample plot**	46–47	39	35	41–49	78
**Recovery time after cessation of use as cropland**,** years**	29	26	22	45	was not plowed

### Productivity

Analysis of phytomass stock in different fractions of plant matter revealed the differences between secondary and virgin steppes, as well as between sample plots with different agricultural use methods. The weight of all fractions of plant matter was the largest in virgin steppe (SP 6) (Table 4). In the restored steppes, the weight of above-ground phytomass and mortmass were the largest in SP 5 with occasional grazing and in SP 2 with occasional haymaking, whereas the lowest values of these indicators were in SP 4 with heavy grazing. The root phytomass in virgin steppe (SP 6) was at least 160 or more percent higher than in all sample plots of restored steppes, which do not differ too much in this indicator despite differences in recovery time and grazing intensity.

**Table 4:** Biomass stock in different fractions of plant matter (g/m^2^) in the sample plots observed in 2023.

No. of sample plot	Method of agricultural use	Above-ground phytomass, g/m^2^	Mortmass, g/m^2^	Root phytomass, g/m^2^
**Secondary steppes**				
2	occasional haymaking	143.0 ± 1.9	175.0 ± 2.8	920.8 ± 73.3
3	moderate grazing	121.2 ± 10.1	113.1 ± 10.3	923.2 ± 58.6
4	heavy grazing	58.5 ± 6.3	20.9 ± 3.0	860.6 ± 70.3
5	occasional grazing	189.1 ± 11.3	285.3 ± 27.1	830 ± 113.3
**Virgin steppe**				
6	occasional grazing	274.1 ± 14.6	373.9 ± 46.5	1543.7 ± 217.6

In 2024, repeated studies of the above-ground phytomass and mortmass showed that this indicator increased significantly in a wetter summer. Compared with the data for 2023 with a normal amount of summer precipitation, in 2024, the above-ground phytomass increased by 298% (from 121.2 ± 10.1 to 362.74 ± 79 g/m^2^) on SP 3 and by 193% (from 274.1 ± 14.6 to 529 ± 48.2 g/m^2^) on SP 6.

The study of the above-ground phytomass of communities in SPs 7–10 located in different parts of the same slope showed that the differences were statistically insignificant between SPs 9 and 10, as well as between SP 7 and SP 8 (Table 5). At the same time, the above-ground phytomass at the foot and in the lower part of the slope (SP 7 and SP 8) was statistically significantly higher than in the communities of the middle and upper parts of this slope (SP 9 and SP 10). The mortmass in the upper part of the slope was lower than in other plots, which is probably due to the fact that dead parts of plants can be washed away by precipitation. The root phytomass was somewhat higher in the sample plot located at the foot of the slope, but this difference was not statistically significant (Table 5).

**Table 5:** Biomass stock in different fractions of plant matter (g/m^2^) in the sample plots observed in 2024.

No. of sample plot	Position of the slope	Above-ground phytomass, g/m^2^	Mortmass, g/m^2^	Root phytomass, g/m^2^
7	Foot	506.7 ± 63.6	155.9 ± 61.1	2128.7 ± 476.9
8	Lower part	510.3 ± 84.5	160.3 ± 42.6	1861.1 ± 314.9
9	Middle part	327.4 ± 37.0	187.7 ± 30.7	1985.0 ± 246.0
10	Upper part	290.7 ± 73.3	35.8 ± 13.0	1968.0 ± 263.1

### Carbon content in biomass and soil

In the sample plots studied in 2023, the carbon content in all fractions of plant matter did not differ significantly and seems to be did not depend on either the floristic composition of the plant communities or on differences in agricultural use methods. However, in SP 2 with episodic haymaking, the carbon content was slightly higher in the above-ground phytomass and lower in the mortmass (Table 6). In 2024, the carbon content in the roots was statistically higher in the sample plots located in the lower part and at the foot of the slope than in the sites located in the upper and middle parts of the slope.

**Table 6:** Carbon content of different fractions of plant matter in the slightly dry year 2023. The SOC content in the plots varied from 2.8 to 6.8% and according to the scale^30^ cover the following categories: “average” (2.3–3.5%), “high” (3.5–5.8%) and “very high” (> 5.8%). The phosphorus content ranged from 37.0 to 59.0 mg/kg and belongs to “low” (21–50 mg/kg) and “average” (50–100 mg/kg) categories; the potassium content ranged from 84.4 to 196.8 mg/kg or considered as “very low” (< 100 mg/kg) and “low” (101–200 mg/kg) categories (Table 7).

No. of sample plot	Method of agricultural use	Above-ground phytomass, g/m^2^	Mortmass, g/m^2^	Root phytomass, g/m^2^
**Secondary steppes**				
2	occasional haymaking	43.1 ± 0.1	36.0 ± 0.5	34.8 ± 0.6
3	moderate grazing	42.4 ± 0.1	39.3 ± 0.3	36.7 ± 0.4
4	heavy grazing	42.0 ± 0.1	40.3 ± 0.6	34.6 ± 0.4
5	occasional grazing	42.7 ± 0.1	38.9 ± 0.4	36.1 ± 0.4
**Virgin steppe**				
6	occasional grazing	42.4 ± 0.2	38.5 ± 0.8	35.1 ± 1.0

**Table 7:** Organic carbon content and stocks in typical Chernozem in 0–30 cm layer. The soil texture of all plots (except for SP 7 with loam) belongs to sandy loam. The coefficient of soil structure (Ks) ranged from 1.06 to 3.18, thus, soil structure was classified^37^ as “excellent” (Ks > 1.5) and “good” (1.5–0.67). Soil aggregate stability was classified as “excessively high” (Ksas > 0.75) for all plots and ranged from 0.81 to 0.92 (Table 8).

No. of sample plot	Method of agricultural use	Organic carbon content, %	Carbon stocks, t/ha
1	arable land	3.5 ± 0.1	108 ± 3
**Secondary steppes**			
2	occasional haymaking	6.7 ± 0.1	199 ± 4
3	moderate grazing	6.7 ± 0.1	201 ± 3
4	heavy grazing	4.3 ± 0.1	141 ± 2
5	occasional grazing	5.8 ± 0.1	181 ± 4
**Virgin steppe**			
6	occasional grazing	3.6 ± 0.1	107 ± 3

**Table 8:** Organic carbon, phosphorus and potassium content of soil on a slope in the 0–30 cm layer (2024).

No. of sample plot	Position of theslope	C_org_, %	*P*_2_O_5_, mg/kg	K_2_O, mg/kg
7	Foot	6.7 ± 0.4	50.8 ± 14.6	196.8 ± 8.8
8	Lower part	5.5 ± 0.1	59.0 ± 15.8	142.0 ± 9.6
9	Middle part	4.4 ± 0.1	41.4 ± 3.1	84.4 ± 4.3
10	Upper part	2.8 ± 0.2	37.0 ± 0.5	111.8 ± 13.5

## Discussion

Compared with secondary steppe, the virgin meadow steppe of the study area is characterized by higher indices of species diversity and species saturation per unit area, as well as a higher proportion of species typical for regional meadow steppes. The proportion of weeds and weed-meadow species in the total species richness is only 13% in the virgin steppe, whereas in restored steppes, these species make a significant contribution – from 23% in the sample plots of the gentle agricultural use (occasional haymaking or grazing) to 43% on the sites with intensive grazing. The time after the cessation of plowing does not play a significant role in the recovery of the composition and structure of the virgin steppe community if the secondary steppe is under intensive grazing.

Under heavy grazing, steppe plants are replaced by inedible, trampling-resistant plants, and this may lead to the formation of a sagebrush-grass community that is very different from the climax meadow steppe of the study area. In addition to mechanical damage (nibbling and trampling), heavy grazing can indirectly alter the composition of grass species by compacting the soil and reducing water availability^39,40^. This reduces above-ground phytomass, leaf area, and light absorption^44,45^. At the same time, moderate grazing is favorable for the restoration of the original near natural steppe vegetation^46^.

In the restored steppes, the above-ground phytomass and mortmass were highest in the sample plots of the gentle agricultural use, while the lowest values for these parameters were in the intensively grazed area. The root phytomass of the virgin steppe community was at least 160% or higher than in the restored steppes. In the secondary steppes under different grazing intensities, the parameters of the root phytomass do not differ much. These results correspond with the data on the secondary steppes of Tuva (southeastern Siberia), where the root phytomass was two times lower than in virgin steppes, while the above-ground phytomass parameters were similar^47^. Since the root phytomass in the upper soil layer increases with the age of the fallow land, which is associated with changes in the plant community composition and improvement of mineral nutrition due to the decomposition of accumulating litter^48^, it is possible to predict a gradual increase in the root phytomass of the secondary steppes of the study area during succession, but it is not yet possible to estimate the time of full recovery.

Thus, in the study area, quite a long succession time of 29–45 years after the cessation of plowing is not enough for the full recovery of the overall species composition, as well as the root phytomass typical for the virgin steppes. These results are close to the data from other regions. For instance, secondary steppes formed on the 30-year-old fallows in the Tuva may have the main characteristics of virgin steppes, especially dominant species, but differ significantly by low floristic similarity indexes and most of the phytocoenotic characteristics^17^. In the secondary steppes on fallow lands of Ukraine, the overall species composition was successfully restored in the secondary steppes ca. 50 years after abandonment, and the share of steppe habitat specialists was similar to the virgin steppe only in the field abandoned for ca. 97 years^49^.

According to Pineiro et al.^43^ and McSherry and Ritchie^50^ overgrazing can reduce SOC content. This result is consistent with literature that grazing reduces root carbon content in a zone with moderate humidity (at rainfall levels of 400–850 mm/yr)^44^.

The above-ground phytomass reserves are also greatly influenced by the position of the steppe community in the relief. Data on the above-ground phytomass reserves in the virgin meadow steppe communities (SPs 7–10) varied greatly depending on the location in different parts of the slope: at the foot and in the lower part, this indicator was about 500 g/m, and in the upper part – 290 g/m. According to the literature, excessive grazing in the middle and especially upper parts of the slopes leads to erosion, soil degradation and loss of fertile topsoil, and therefore the carbon content in the upper part of the slope did not differ from its content in arable land. In general, this is typical for habitats with low precipitation and high evaporation^15,51^. This is consistent with our case, for example, the SOC content on a virgin plot located in the middle part of a steep slope (SP 6) was comparable to that of an arable land. It is worth noting, that the most suitable flat and fertile areas were selected for plowing, while steep slopes were not used as croplands. On the one hand, on unploughed (virgin soils), the development of erosion should be minimal, but with annual erosional runoff, the plant mortmass does not have time to decompose/humify and is partially washed away down the slope.

**Fig. 3:**
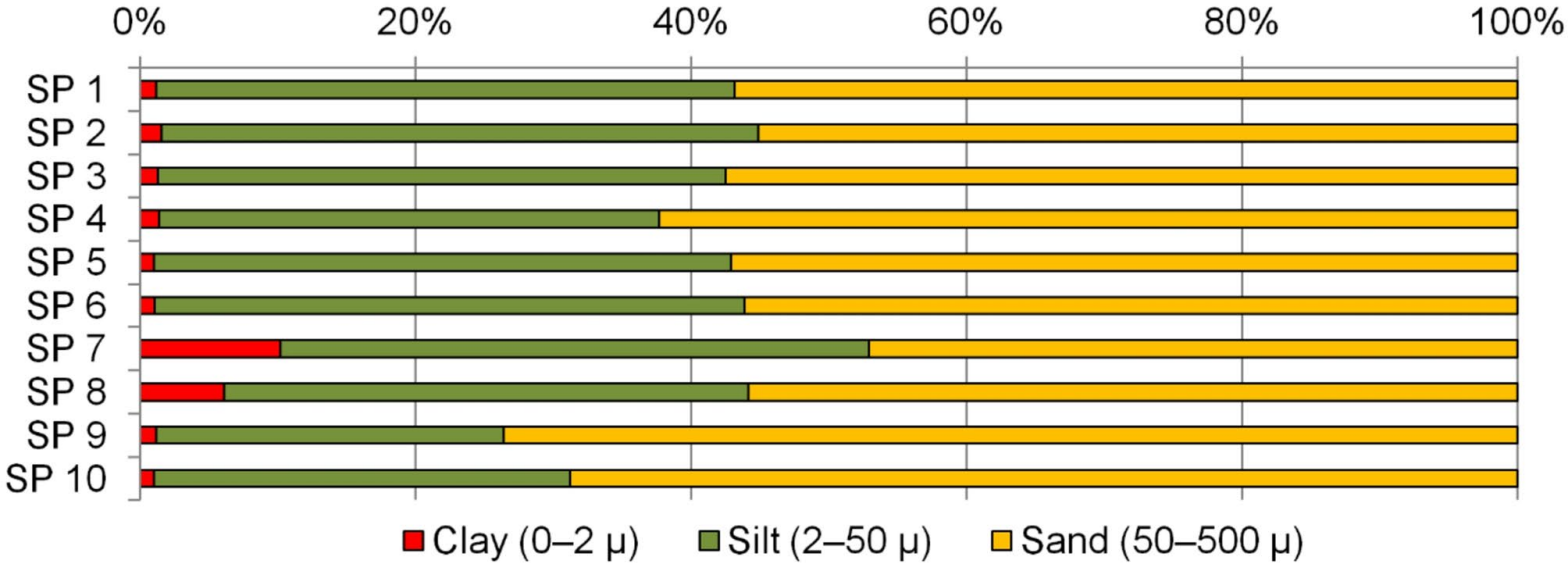
Granulometric composition of soils (0–30 cm).

In general, the content and reserves of SOC in the soil do not directly correlate with the reserve of biomass, which was highest in plots of 2023 and in SP 6 (middle of the slope) (Table 6). The SOC content in the soil was highest not in the uncultivated areas but in the leveled areas of the secondary steppe: SP 2 (episodic haymaking) and SP 3 (moderate grazing). Moreover, the carbon content in these areas was almost twice as high as in the arable land and did not differ from the SOC content at the foot of the slope (Table 7). The content of nutrients in the soil also varied depending on the position of the plot on the slope. The highest values of C_org_, available phosphorus and exchangeable potassium were found at the foot and at the bottom of the slope. This is most likely due to erosion processes. The study area, like the Southern Cis-Urals as a whole, is considered a region susceptible to erosion: potential and maximum possible erosion rates vary from 10 t·ha^−1^·yr^−1^^52^ and 20 t·ha^−1^·yr^−1^^53^ to 60 t·ha^−1^·yr^−1^^54^, or approximately 0.6 to 5 mm per year, respectively. In modeling rainfall erosion of meadow chernozem soils with a similar steep slope (~ 12°) with a rainfall intensity of 6 mm·min^−1^ and a duration of about 50 min, soil losses reached 50 t·ha^−1^^55^, while the washed-off sediments also contained more organic matter and nutrients. It is known that the upper fertile soil layer, as well as the upper and middle parts of the slope (where the kinetic energy of the runoff gains the greatest destructive force), are subject to erosion, and soil particles (in particular the silt and clay fractions^56^) enriched with nutrients are washed away with the water-erosion flow^57^. Thus, the content of C_org_ and potassium in the upper and middle parts of the slope was approximately 2 times lower than at the foot or lower part of the slope, and the phosphorus 20–37%. At the same time, on the elevated relief elements (SP 9 and SP 10), the sand content (Fig. 3) was high (70–75%), and in the accumulation zone (SP 7 and SP 8), fine fractions (clay and silt) prevailed, which confirms the hypothesis of the washout of such fractions rich in nutrients from the slope. No significant differences in particle size distribution were found between the arable land areas and those subject to grazing of varying intensity, and the soils of these areas are characterized as sandy loam. The sand content was somewhat higher in the area with heavy grazing, which indicates the development of pasture digression in this area, i.e., an increase in degradation processes, in particular water and/or wind erosion^58^.

**Fig. 4:**
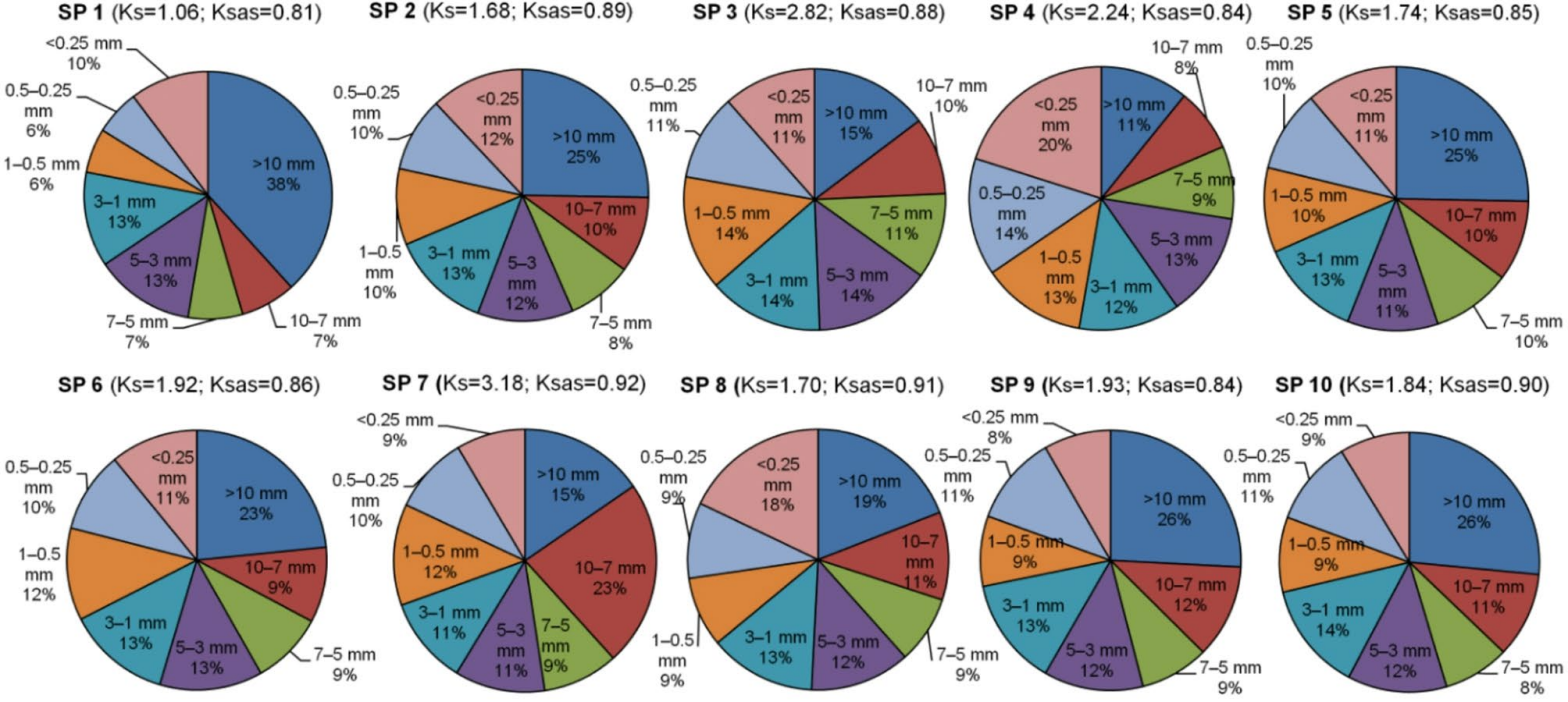
Structural and aggregate composition of soils (0–30 cm).

Reduced grazing and soil disturbance have a positive effect on the restoration of degraded grasslands and the improvement of carbon sequestration and soil organic matter^59–61^. Moderate grazing and occasional haying in 2023 had a positive effect on soil carbon sequestration, similar to high-intensity grazing for a short period^62^, which is associated with the stimulation of root growth^63,64^. The development of root and plant biomass (including projective cover) also affected the soil structure. If on arable land the share of agronomically valuable aggregates (Σ of 0.25–10 mm) was 51%, then on pasture areas their share varied in the range of 65–74% (Fig. 4). Despite the presence of vegetation, grazing led to the destruction of aggregates, especially large ones; for example, blocky/lumpy aggregates (> 10 mm) prevailed on arable land, while their share was not high in grazed areas and decreased with increasing grazing intensity. At the same time, the lowest share of blocky fractions and the highest share of dusty were found in the intensively grazed area. This was undoubtedly due to frequent mechanical action/destruction of soil/aggregates by cattle. Obviously, the “best” structure and high water resistance of aggregates were on the virgin area at the foot of the slope (SP 7), but in the sloping areas (SPs 7–10) it deteriorated as erosion developed. As is known, the quality of the soil structure and the water resistance of aggregates are negatively affected by agricultural activities (plowing, grazing, etc.), as well as erosion processes^65–67^.

Precipitation variability significantly affects the functioning of meadow steppes, as well as other water-limited systems^68,69^. Comparison of data for 2023 with normal moisture conditions and 2024 with high spring-summer precipitation showed that in 2024, above-ground phytomass stock increased almost twofold in the virgin steppe with episodic grazing and threefold in the secondary steppe under the influence of moderate grazing. The average stocks of above-ground phytomass are 350 and 370 g/m^3^ in the meadow steppes of Siberia and European Russia, respectively^70,71^. Compared with these data, the above-ground biomass stocks in the virgin steppe of the study area were lower in 2023 (274 g/m^2^) and significantly higher in 2024 (529 g/m^2^). The above-ground phytomass stocks of the secondary steppes of the study area approached the data on meadow steppes of other Russian regions only in wet weather conditions of 2024.

It is widely accepted that increased soil moisture improves the carbon cycle^72–74^. The change in soil water content (SWC) with precipitation closely corresponds to changes in precipitation volume^75–77^ and extreme events^78–80^.

Increased precipitation in 2024 did not have a significant effect on SOC content, since it was formed over a long time. Thus, differences in annual precipitation patterns may affect carbon cycling processes in steppe ecosystems^81–83^. However, the effects of precipitation changes on soil moisture are still poorly understood, and thus additional quantitative data are needed^69,82–85^.

## Conclusions

In the Bashkir Cis-Urals, the secondary meadow steppes on fallow lands abandoned for ca. 20–45 years are close to virgin steppes in terms of the composition of dominants, but are characterized by low floristic diversity, the proportion of specialist species, and the root phytomass. Under gentle agricultural use (occasional or moderate haymaking or grazing), the succession goes towards the virgin meadow steppe with the restoration of the organic carbon content in the soil. Intensive grazing slows down the restoration and reduces the organic carbon content in the soil. Thus, by regulating the intensity of grazing, it is possible not only to increase the productivity of meadow steppes on relatively leveled areas, but also to increase the deposition of carbon by the soil. Meadow steppes, confined to the upper and middle parts of hillsides, have high above-ground phytomass stock but contain less carbon due to water erosion. In connection with the predicted climate aridization, an increase in the frequency of droughts while maintaining periodic years with in-creased precipitation, additional research is needed to assess the productivity and level of carbon sequestration in the context of climate change.

## Data Availability

The datasets generated during and/or analysed during the current study are available from the corresponding author on reasonable request.
